# Reports on the Hospitals of the United Kingdom

**Published:** 1909-12-11

**Authors:** Henry Burdett


### REPORTS
ON THE
### Hospitals of the United Kingdom.

By SIR HENRY BURDETT, K.C.B., K.C.V.O.

### XIII.—THE LEICESTER INFIRMARY.

This institution was established in 1771, and it has made great strides since we inspected it about twenty-five years ago. There are few, if any, county hospitals where the responsibilities of the managers are as great as at Leicester. Leicester is the one county in England where the Infirmary's influence has been so strong as practically to exclude the Cottage Hospital movement, though the population exceeds half a million.

This commanding influence is due to the men, and the wonderful spirit they have brought to bear upon the work, who have been responsible for the administration of its affairs. At the present time the Leicester Infirmary is specially fortunate in its chairman, Alderman Sir Edward Wood, J.P., who has thrown himself heart and soul into the reconstruction scheme, which has cost £100,000. Owing to the munificence of Sir Edward Wood and to his really wonderful initiative, this fund in seven years has been raised to £96,000. Splendid efforts like those made by Sir Edward deserve every acknowledgment and the fullest and highest public recognition. It is impossible to spend some hours in a close inspection of this hospital without realising that the resident staff is highly competent and deeply interested in their work. The house-governor and secretary, Mr. Harry Johnson, is one of the most efficient and up-to-date of superintendents, and we have known and appreciated from the outset of his career the excellence of the work done by the lady superintendent, Miss Gertrude Rogers, who has been attached to this institution for the long period of twenty-six years.

### Recent Reconstruction Works.

To enable the hospital world to grasp the full measure of Sir Edward Wood's work, we set forth a summary of the improvements now completed:—

The entire reconstruction of the drainage system.

The removal of the old boiler-house, and the erection of a new one with a pump-room, together with a disinfector and refuse destructor on a new and suitable site.

The thorough reconstruction of six very old wards in the north-east and north-west portions of the building, together with the addition of separate sanitary blocks detached from the wards.

The removal of the out-patient department to a new site, on which has been erected a large and complete building, referred to below.

The reconstruction of the kitchen and culinary department.

Next, the Board had to face the want of adequate accommodation for in-patients, which resulted in an expenditure of £26,500, whereby 100 additional beds were provided in three new wards. The hospital laundry was reconstructed at a cost of £2,500, and the new nursing home, now completed, will cost altogether, with the furniture, some £21,500. In this connection it is just to mention, as a tribute to the influence and the good work of the nursing staff, that Miss Rogers organised a bazaar on behalf of the home, which realised the magnificent sum of £4,510.

There remains the relatively small outlay which will have to be found in order to bring the children's hospital and the mortuary block up to date. It affords us great pleasure to endorse with conviction Sir Edward Wood's opinion that, when this work has been provided for, Leicester will possess a well-equipped building which should adequately meet the hospital needs of the counties of Leicester and Rutland for years to come. What is more, as we have already intimated, the present excellent condition of the hospital in all departments, and the evidence our inspection has supplied of the efficiency of the whole staff, afford guarantees to the public which should make them welcome the opportunity to subscribe the relatively small sum which is still required to complete the work. That sum amounts, according to our estimate, to £15,000 to defray the balance of the cost of the nursing home and to provide funds for the reconstruction of the children's and mortuary blocks.

### How to Provide Income.

There is also immediate and urgent need for an additional income of £3,000. This additional revenue ought to be speedily forthcoming by a revision of the present privileges of subscribers. The present privileges are altogether too large, and they are infinitely greater than the majority of modern hospitals grant. The report does not contain, so far as we can find, the cost of each in- and out-patient for 1908. The average cost in 1907 of each in-patient may be taken at £4 16s., and that of each out-patient, roughly, at 2s. Each subscriber of one guinea is entitled to one in-patient and two out-patient tickets. It therefore appears that each subscriber for each subscription of £1 1s. receives £5 in value as represented by the actual cost of his patients to the infirmary. Such a system as this, as we have often said before, is neither good business nor sound philanthropy. The committee of the Leicester Infirmary may, in our view, properly follow the example of the Shrewsbury Infirmary by revising their scale of privileges so as to put it on a more businesslike and better footing. If this is done, seeing that the annual subscriptions at present yield a revenue of £3,764, the additional

£3,000 a year should be speedily forthcoming. A similar revision at Shrewsbury Infirmary produced excellent results. As a further stimulus to revenue, the Committee would be well advised to consider the question of affording every patient the privilege of paying something for his maintenance, according to his means. They might further provide certain pay wards under proper regulations for the protection of the medical profession and the public, which should yield a definite revenue each year to the hospital. We might perhaps suggest that a further means of raising revenue would be to appoint a small committee to reconstruct the annual report, with the object of publishing an account each year, showing the annual relief afforded to the inhabitants of each parish or township, the amount of revenue received from the inhabitants of each such place, and the balance in favour of or against its population, so as to bring home to the people the actual amount of benefit they are receiving, and the actual claim which the infirmary has upon them as individuals for increased monetary assistance. We think, too, seeing the general excellence of the present buildings and their many attractive features, that additional interest might be lent to the report by the inclusion of a number of photographs each year and a plan of the whole hospital.

The Nursing Department.

Another special feature is that Leicester Infirmary, which will soon contain 270 beds and has several able consultants on its staff, is not a clinical hospital. The absence of students and the excellence of the surgical and medical work make this hospital ideal as a training school for nurses. Yet with a staff of eighty nurses the accommodation for them has been as out of date as anything to be found in the least advanced hospitals. The committee have exhibited the most progressive spirit in regard to other departments. Recognising the imperativeness of a new nurses' home, they decided last year to put the matter in hand at once. With their usual spirit they have sanctioned the erection of an exceptionally spacious and promising home for one hundred nurses, which is to be ready for occupation by the end of 1909. Then the nurses of the Leicester Infirmary should find themselves fortunate indeed. This will become evident when we publish a full plan and description of the New Nurses' Home.

Good Points.

We made a thorough inspection of the hospital and found many things that were admirable. Even the old wards as converted are by no means bad, though the ward offices and some of the fittings, notably the slop sinks, should be replaced as opportunity offers. The latest pavilion contains wards with thirty beds each. These wards are hygienically very good, the ventilation is complete, and the temperature of the wards can be kept at a low degree, which is a great advantage. So airy and light and spacious are these wards that they ought to prove ideal for surgical cases especially. Of course the size of the wards puts an extra strain on the staff, and particularly on the nurses. Patients who gain hygienically find the ward troublesome, as the lavatories are at one end, and any patient occupying a bed below the middle of the ward has to walk so far as to prove fatiguing in the days of early convalescence. We should say that there can be no doubt that wards with about twenty beds are the best on the whole for a modern hospital.

The out-patient department has been well thought out, and in this and other portions of the establishment the Leicester Infirmary is in advance of its neighbours, although some of them are clinical hospitals.

The laundry is large, and is well fitted with machinery. It turns out 6,000 pieces a week. The drying chambers are especially good, and the colour of the linen was excellent also.

A Useful Officer.

The Infirmary has the advantage of a young and capable dispenser, who is doing excellent work. He makes all the galenicals, and is saving from £30 to £40 a year by collecting the spirit in the cylinders used in the theatres and re-distilling it. He has also organised a separate dispensary and separate order system for the supplies to the out-patient department without putting the Infirmary to any increased cost for service, or in any way. This proves the truth and force of the contention urged in The Hospital, that, providing the authorities have the will, they can readily provide the means whereby the cost of each out-patient and in-patient can be exactly ascertained without extra expense.

The Children's Block.

The Children's Hospital, as it is called, is a separate pavilion improperly built on to one of the older wings of the infirmary. Although comparatively new, the whole of this building needs dusting out; that is to say, the divisions which shut off the end of the wards from the larger portion of them should be removed, and the whole of the ward kitchen and lavatory accommodation should be reconstructed, remodelled and partly refitted. This Children's Hospital does not compare favourably at present with other portions of the Infirmary.

The Mortuary Buildings.

The mortuary block is not a credit to this institution; it is badly planned, and one room in it is dirty and objectionable, being used to repair bicycles, etc., a most improper arrangement, seeing that it can only be entered through the post-mortem room or through the mortuary and post-mortem room combined. It contains no mortuary chapel, and patients' friends have to view the body of a relative in the section where all bodies are placed together on biers. We should hope that the spirit of reverence for the dead and of sympathy for the living is strong enough to render it certain that the mere mention of these facts will at once produce the necessary funds to secure the erection of a properly constructed mortuary unit. We must add that the post-mortem room, in regard to both fittings and cleanliness, left much to desire.

---

**The Nottinghamshire General Hospital** will be reported on next week.

